# Early Association of Prosodic Focus with *alleen* ‘only’: Evidence from Eye Movements in the Visual-World Paradigm

**DOI:** 10.3389/fpsyg.2016.00150

**Published:** 2016-03-11

**Authors:** Iris Mulders, Kriszta Szendrői

**Affiliations:** ^1^Utrecht Institute of Linguistics OTS, Utrecht UniversityUtrecht, Netherlands; ^2^UCL Linguistics, Psychology and Language Sciences, University College LondonLondon, UK

**Keywords:** focus, semantics, marked stress, prosody, incremental language processing, eye tracking, visual world paradigm, anticipatory eye movements and predictions

## Abstract

In three visual-world eye tracking studies, we investigated the processing of sentences containing the focus-sensitive operator *alleen* ‘only’ and different pitch accents, such as the Dutch *Ik heb alleen SELDERIJ aan de brandweerman gegeven* ‘I only gave CELERY to the fireman’ versus *Ik heb alleen selderij aan de BRANDWEERMAN gegeven* ‘I only gave celery to the FIREMAN’. Dutch, like English, allows accent shift to express different focus possibilities. Participants judged whether these utterances match different pictures: in Experiment 1 the Early Stress utterance matched the picture, in Experiment 2 both the Early and Late Stress utterance did, and in Experiment 3 neither did. We found that eye-gaze patterns start to diverge across the conditions already as the indirect object is being heard. Our data also indicate that participants perform anticipatory eye-movements based on the presence of prosodic focus during auditory sentence processing. Our investigation is the first to report the effect of varied prosodic accent placement on different arguments in sentences with a semantic operator, *alleen* ‘only’, on the time course of looks in the visual world paradigm. Using an operator in the visual world paradigm allowed us to confirm that prosodic focus information immediately gets integrated into the semantic parse of the proposition. Our study thus provides further evidence for fast, incremental prosodic focus processing in natural language.

## Introduction^1^

### Prosodic Focus and Contrast: Pragmatic Effect

Focus is an important^1^ information-structuring device. It occurs in every utterance, and it signals to the hearer the most prominent part of the utterance: what is new, or what is contrasted or highlighted. In many languages including English and Dutch, it is marked by prosodic prominence, specifically, with a pitch accent. Focus processing is crucial for comprehension of utterances in context. To illustrate this, we can consider what makes a question–answer pair pragmatically felicitous. Capitals indicate prosodic stress and corresponding pitch accent throughout. (1b), with prosodic accent on the direct object ‘some tea’ is a felicitous answer to the question in (1a), while (1c) with prosodic accent on the indirect object ‘to the woman’ is not. (Rather, it would be a felicitous answer to a different question, namely ‘Who did you give some tea to?’) This is because the question in (1a) asks for information about the object that was given to the woman, and thus expects the responder to prosodically highlight the direct object in their response.

(1) a. What did you give to the woman?
  b. I gave some TEA to the woman.
  c. #I gave some tea to the WOMAN.

Prosodic focus can also play an important role in determining the felicity of utterances in a non-linguistic context. An utterance like (2), with contrastive accent on the modifying adjective, is only felicitous in a context where not just green balls, but balls of some other color are also present. The pragmatic function of the accent placement here is contrastive.

(2) Give me the GREEN ball.

Given the importance of prosodic accent placement for information structuring, many studies have tried to uncover the effect of prosodic prominence on language processing. Eberhard et al. (1995) were the first to report an experiment involving a reference resolution task in a real world setting involving prosodic prominence. The instructions involved either contrastive or neutral stress (*Touch the LARGE/large blue square*) on modifying adjectives in two different visual contexts. The results showed that in the contrastive stress condition, the latency of eye movements to the target referent was significantly shorter in the setting where contrastive stress was informative (i.e., in a context with a large and a small blue square) than in the uninformative setting (i.e., in a context with an additional pair of large and small matching objects). The eye movement latency was also shorter in the contrastive stress condition compared to the unstressed condition. So, contrastive stress facilitated reference resolution (but cf. Arnold, 2008).

The facilitatory effect of the contrastive L+H^∗^ accent in English reference resolution tasks in contrastive contexts has been further supported in a series of experiments by Ito and Speer (2008, 2011). Here participants heard pairs of instructions like *Hang the blue ball* with a second instruction following bearing either neutral or contrastive stress, e.g., *Next, hang the* green/*GREEN ball*. In addition to confirming the processing advantage of contrastive accents on the modifying adjective when used in a contrastive context, Ito and Speer also demonstrated that the use of such accents leads to anticipatory looks to the previously mentioned entity type (i.e., balls) and to ‘garden path’ effects if used in infelicitous contexts (e.g., *blue angel* followed by *GREEN ball*). They thus demonstrated early interpretation of contrastive prosody.

Note, however, that in all these experiments, the reference resolution task can also be carried out without the presence of the contrastive accent. In other words, were the instructions read out with a different intonation, the reference resolution task could still be carried out correctly. The presence of the contrastive accent is facilitatory and its absence informative, but ultimately, it only has a pragmatic effect: it does not contribute to the sentence meaning directly as it does not change the truth conditions of the sentence.

### Prosodic Focus and *Only*: Semantic Integration

Prosodic focus placement is not only relevant for pragmatic felicity of certain utterances in linguistic or non-linguistic context. Sometimes the position of the prosodic focus within the utterance directly contributes to the semantic meaning of the utterance. Sentences that involve the operator *only* are an example of this.

(3) I only gave some tea to the woman.

In sentences involving an operator, like *only*, the prosodic focus placement is not only relevant for pragmatic felicity of the utterance in context. Rather, the position of the prosodic focus within the utterance directly contributes to the semantic meaning of the utterance. In fact, the correct semantics cannot be determined without accentual information. So, presented in writing, (3) is ambiguous; its meaning depends on its accentuation pattern. The ambiguity can be resolved by prosody, as in (4).

(4) a. I only gave some tea to the WOMAN. = The only person I gave some tea to was the woman.
  b. I only gave some TEA to the woman. = The only thing I gave to the woman was some tea.

In (4a), with pitch accent on the indirect object, *only* associates with the stress-bearing indirect object, *the woman*, while in (4b), with stress on *tea*, *only* associates with the direct object, *some tea*. Accordingly, linguistic theories agree that the different interpretations in (4a) and (4b) are an indirect result of the two stress patterns; they arise because the operator *only* is focus-sensitive, meaning that it associates in its interpretation with the focus of the utterance (Horn, 1969; Krifka, 1992; Rooth, 1992). Focus, in turn, is determined by main stress and corresponding pitch accent in English (Chomsky, 1971) and Dutch.^2,^^3^

In order to be able to consider the psycholinguistic aspects of processing *only-*sentences, we need to understand in a little more detail how the semantic meaning of such sentences is determined. Utterances with *only* can be decomposed into two conjoined propositions (Horn, 1969; Krifka, 1992; Rooth, 1992). The first conjunct, (5b) and (6b), respectively, correspond to the meaning of the proposition without *only.* This is called the ‘presupposition’ or the ‘non-focal meaning component’. This part of the meaning is shared by the two utterances with indirect object and direct object stress (5a and 6a). The second conjunct is entailed by the original *only*-sentence. It expresses the fact that the presence of *only* has the effect that the proposition does not hold for any other relevant alternatives. This part of the meaning is called the ‘assertion’ or the ‘focal meaning component’ (5c and 6c).

(5) a. I only gave some tea to the WOMAN.
  b. Non-focal meaning component:
    I gave some tea to the woman AND
  c. Focal meaning component:
    For all x [x ≠ the woman], I did not give some tea to x.
(6) a. I only gave some TEA to the woman.
  b. Non-focal meaning component:
    I gave some tea to the woman AND
  c. Focal meaning component:
    For all y [y ≠ some tea], I did not give y to the woman.

As we can see from (5c) and (6c), it is the focal meaning component that bears the semantic difference between the two utterances with different prosodic accent placement. The non-focal meaning component is the same. So, it is the focal meaning component that we need to target in our psycholinguistic investigations.

It helps to understand that the focal meaning component is in fact a set of conjoined propositions. In the case of (5c), we can spell it out as in (7a), while (7b) corresponds to the focal meaning component of the direct object stress utterance, (6c). The exact number of alternatives in each assertion set is determined by the actual context of the utterance. So, for instance, in (7a) we used a context where a man and a boy are present in addition to the woman, while in (7b) we used a context where some coffee and biscuits were available alongside the tea.

(7) a. {I didn’t give any tea to the man AND I didn’t give any tea to the boy}
  b. {I didn’t give any coffee to the woman AND I didn’t give any biscuits to the woman}

Let us now turn to the psycholinguistic characteristics of processing *only-*sentences. By studying the auditory processing of sentences like (3) we can investigate how fast prosodic focal information gets integrated into the semantic parse of the utterance. In other words, as soon as we can detect evidence that people can distinguish the meaning in (4a) from the meaning in (4b) in online auditory comprehension, we can conclude that they have processed the prosodic focal information and integrated that information into the semantic parse of the utterance. Evidence of this can come from evidence of the participants considering the focal meaning components in (5c) and (6c) or their equivalent set of propositions in (7a) and (7b).

There are two possibilities regarding the timing of this computation. First, it is possible that the integration of focal prosody information is very fast and incremental. This would match the Ito and Speer (2008) findings about contrastivity. If so, one should see evidence of the non-focal meaning component being considered at the earliest possible point. Given the semantics of *only-*sentences described above, the earliest point that the focal meaning component (i.e., 5c and 6c) can be considered is when the proposition is complete. This is even true of utterances with early stress on the direct object, as in (6a). This is because even though in such utterances the prosodic focus is available earlier, during the direct object, in order to integrate that information into the semantic parse and compute the non-focal, and focal meaning components, it is necessary to know the whole proposition, i.e., the verb and the indirect object.

The second possibility is that semantic integration of prosodic focus is considerably slower than pragmatic effects of contrastivity. Perhaps due to the complex nature of the calculations involved in the semantics of *only*-sentences (i.e., non-focal and focal meaning components), it is possible that evidence of the non-focal and focal meaning components being considered would not emerge until well after the utterance offset, during wrap-up processing. Perhaps pragmatic effects of contrastivity would be manifest, as found by Ito and Speer (2008), at the point of the occurrence of the prosodic focus itself. But semantic integration of the prosodic focus information would be delayed.

A number of reading studies have been done involving *only-*sentences. Paterson et al. (2007) compared reading times for dative sentences (and also double object constructions) where the position of the focus particle varied between a pre-direct object position (e.g., *Jane passed only the salt to her mother*) and a pre-indirect-object position (e.g., *Jane passed the salt to only her mother*). In these constructions *only* associates with the immediately adjacent noun phrase. In terms of the semantics of *only*-sentences discussed above, this means that the focal meaning components for the test sentences were *Jane didn’t pass anything else to her mother* and *Jane didn’t pass the salt to anyone else*, respectively. Accordingly, they used congruous vs. incongruous replacives as continuations to the sentences (such as *but not the pepper / but not her father*) to determine whether participants are sensitive to the placement of the focus particle when creating contrasts. This is based on the expectation that if participants compute the relevant focal meaning component by the time they encounter the replacives, they would find them incongruous if they are mismatched. They found that the position of *only* evoked the expected focus effect on-line (see also Sauermann et al., 2013). This, however, manifested itself in longer reading times for the postreplacive region, rather than the replacive region itself. Paterson et al. suggested that ‘this delay […] was attributable to the operation of inferential processes to evaluate the congruency of the supplied contrast’ with the focus structure of the sentence (Paterson et al., 2007, p. 1440). Given this delay, it is not possible to determine whether the semantic integration of focus is itself late, or if it happens earlier, but is masked by the delay caused by the inferential process involved in determining the focus in the absence of direct prosodic information.

Another study that involved sentences with *only* is the self-paced reading experiment by Crain et al. (1994), extended by Sedivy (2002) (cf. Paterson et al., 1999; Clifton et al., 2000; Liversedge et al., 2002; Filik et al., 2005). This study investigated variants of established garden-path sentences involving *only*:

(8) a. Businessmen loaned money at low interest were told to record their expenses.
  b. Only businessmen loaned money at low interest were told to record their expenses.

The results indicated that the presence of *only* ameliorates the garden path effect. This effect is consistent with a scenario where participants create a contrast set based on the presence of the operator, prompting them to build the appropriate reduced relative clause structure already before the disambiguating main clause verb *were told* appears. The amelioration of the garden path effect disappears again when a contrast set is given in the discourse. We can take this as evidence that *only* prompts readers to generate a contrast set (if there is none in the context) at some point before the disambiguating main clause verb. But we do not know exactly at what point it happens before then.

Overall, while these reading studies indicate that focus information is used during processing, by their nature reading studies cannot be informative about the disambiguating role of stress, as stress is generally not marked in writing. Furthermore, reading studies typically tap into the analysis that participants make by disconfirming this analysis later on in the text, measuring a resulting slowdown effect at that point; this means that there can always be a gap between the point in time where the analysis was made by the participant and when we detect its effects.

The visual world paradigm can give precise information about the interpretation of the sentence at each point in time during the sentence. Gennari et al.’s (2005, p. 250) measured response times and overall fixation patterns in a visual-world paradigm, using a visual setup involving three people: for instance, a woman, a man and a boy. In the picture, the boy had a glass of milk in front of him, the man had a glass of milk and a cup of coffee. The woman, standing in the background, was holding a tray with a milk carton and a teapot. Participants heard utterances either with neutral stress on the indirect object (like 9a) or with marked stress on the direct object (9b) in a picture verification task.

(9) a. The mother only gave some milk to the *boy*. Neutral stress FALSE
  b. The mother only gave some MILK to the boy. Marked stress TRUE

Gennari et al. (2005) proposed that ‘marked stress is used immediately by the parser to decide which noun phrase bears semantic focus and, therefore, which contrast set should be invoked for sentence interpretation’. In other words, they proposed that focus processing is fast and incremental in *only-*sentences. They reached their conclusion based on their finding that in the Neutral Stress condition, there were fewer correct responses than in the Marked Stress condition (MS: proportion of correct responses 0.84, *SD*: 0.18; NS: 0.70, *SD*: 0.19). Note that this is an indirect reasoning: there could be many reasons why the number of correct responses was lower in the Neutral Stress condition that have nothing to do with the potential early integration of Marked Stress information. They did not find a response time difference between the two conditions (Gennari et al., 2005, p. 254). Note, however, that the expected responses diverged in the two conditions (MS: TRUE, NS: FALSE). It is possible that this influenced response times because it may take longer or shorter to verify a proposition than to falsify it. There was also a qualitative difference between the phonetic salience of neutral and marked stress, which may have boosted participants’ performance in the Marked Stress condition.

Gennari et al. (2005) only report overall proportion of looks on the various entities treating the entire utterance and the time between the utterance offset and the participants’ response as one single time window. They found that the ‘boy’s milk’ draws a significantly higher proportion of looks when it bears contrastive stress (i.e., MS) compared to when it does not (i.e., NS).^4^ However, the different pattern of looks across the conditions can only be interpreted as evidence for early, incremental effect of focus if they are time-locked to the appearance of the prosodic focal information in the auditory input. To establish this, one would need to know not only the overall fixation patterns, as provided by Gennari et al. (2005), but also how the eye movements progress as the sentence unfolds. To sum up, Gennari et al. (2005) found that the number of correct responses was higher in the Marked Stress condition, but no difference in response times. They also found that entities bearing contrastive focus are targeted more by eye gaze if overall looks are considered, raising the possibility that a pragmatic effect of contrast occurs early. They did not investigate the time course of semantic integration of prosodic focal information.

To sum up, we have seen that prosodic focus, as an information structuring device, often has pragmatic effects, i.e., it makes certain utterances felicitous or infelicitous in context. One such effect is contrastivity, which was investigated by Eberhard et al. (1995) and Ito and Speer (2008, 2011). What they found was that contrastive prosody facilitates reference resolution and that the contrastive information is interpreted with respect to the actual context. But focus can also directly contribute to the semantics of the utterance if an operator such as ‘only’ is present in the utterance. In such sentences, one simply cannot compute the full meaning of the utterance without knowing where the prosodic focus is. So in such sentences, focus has a semantic effect, not just a pragmatic one. Semantic integration of focus was investigated by Paterson et al. (2007), but in a reading study, so the position of the focal prosody is only inferred, which may have contributed to the observed delay in the integration of the prosodic focus information into the semantic parse.

### Our Study

We investigated utterances with *alleen* ‘only’ with different focal accent placements in a visual world paradigm, such as the Dutch *Ik heb alleen SELDERIJ aan de brandweerman gegeven* ‘I only gave CELERY to the fireman’ versus *Ik heb alleen selderij aan de BRANDWEERMAN gegeven* ‘I only gave celery to the FIREMAN’. Our objective was to detect the earliest point that participants’ eye gaze patterns give evidence that they consider the propositions that make up the focal meaning component of the utterances with different focal accents. Our study thus reveals how fast different focal accent placements on the arguments of the verb get integrated into the semantic parse of the utterance during auditory comprehension. We carried out three visual-world paradigm experiments to investigate these issues, measuring response times and the time course of eye movements. In Experiment 1 the divergent expected responses (Early Stress: YES; Late Stress: NO) corresponded to the Gennari et al. (2005) study to maximize chances of comparison. In Experiment 2, the visual stimulus was adapted in such a way that the expected response was YES in both conditions. In Experiment 3, the visual stimuli were changed to trigger NO responses in both conditions.

**FIGURE 1:**
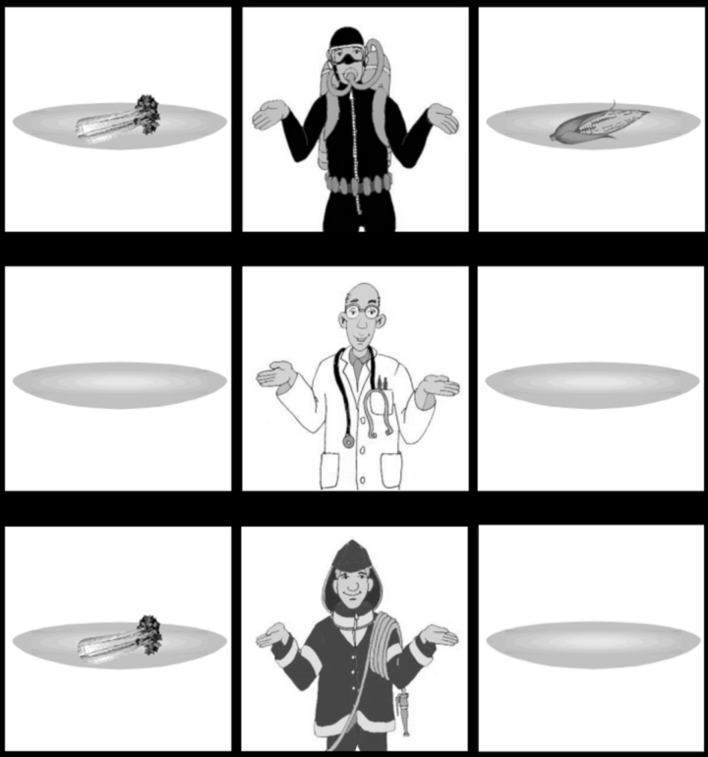
**Example of visual stimulus for Experiment 1**.

Our hypothesis was that if participants integrate prosodic focal information immediately, their looks will reflect the semantic parsing of the utterance during utterance comprehension. The alternative position is that only the pragmatic effect of contrast is fast, while semantic integration of prosodic focus into the parse only appears later, during wrap-up computation. In order to determine the expected looks during sentence verification, let us apply Rooth’s (1992) semantics to the specific example from our experiments to the visual scene seen in Experiment 1, see **Figure 1**.

(10) Early Stress (ES) condition:
  a. Example in English: I only gave CELERY to the fireman
  b. Non-focal meaning: I gave celery to the fireman
  c. Focal meaning: I did not give anything else to the fireman = {I didn’t give x to the fireman AND I didn’t give y to the fireman AND I didn’t give z to the fireman … , where x, y, z, … are objects that could have been given to the fireman in the context}
  d. Potentially relevant entities for verification of focal meaning in visual context: fireman and his objects

(11) Late Stress (LS) condition:
  a. Example in English: I only gave celery to the FIREMAN
  b. Non-focal meaning: I gave celery to the fireman
  c. Focal meaning: I did not give celery to anyone else = {I didn’t give celery to x AND I didn’t give celery to y AND I didn’t give celery to z … , where x, y, z, … are people that a celery could have been given to in the context}
  d. Potentially relevant entities for verification of focal meaning component in visual context: any other person in the picture and their objects
  e. Falsifying proposition in Experiment 1: I gave celery to the diver.
  f. Entities relevant for the falsifying proposition in Experiment 1: diver, diver’s celery

Concerning the time course of the verification procedure the following predictions hold. The earliest point at which the focal or non-focal meaning components can be verified is when the proposition is complete. Given that the verb is predictable in our experiment, we may actually find verification of the focal (and non-focal) meaning component to start at the indirect object directly preceding it. In the ES condition, marked stress and thus focus can be identified earlier: at the point when the direct object is heard. But note that the focal meaning components cannot yet be computed at this point. When the participant hears *I only gave CELERY to the…* the sentence could end in two different ways. Either the indirect object turns out to be *the fireman*, as in our actual example, in which case the utterance matches the picture, or the indirect object could turn out to be *the diver*, in which case the utterance would not match the picture. (In principle, the utterance could also end with the indirect object *the doctor*, but this would constitute an infelicitous utterance. Since the doctor has no celery in the picture, the non-focal meaning component would not be true.) For this reason, we do not expect a difference in response times across the conditions. In terms of expected number of correct responses, we do not expect a difference between the conditions either. Both the ES and LS conditions contain prosodically marked, contrastive stress associated with *only* so there is no reason to expect that either condition would be easier to perform than the other.

Given the basic linking hypothesis that auditory input guides visual attention, one would expect looks to target the entities mentioned in the utterances, i.e. the fireman and any celery when these words are heard. Looks to these entities could also reflect verification of the non-focal meaning component, which directly corresponds to the utterance without *only*.^5^

The main goal of this paper is to investigate the effects of prosodic focal accent on the direct object versus the indirect object in *only*-sentences. For this reason, we will be primarily interested in the looks that can be attributed to the different focal meanings. The looks required to verify the focal meaning components differ across the two conditions. Let us take the ES condition first. In order to verify *I didn’t give anything else to the fireman*, looks need not shift away from the fireman and his plates. Participants simply need to check that the fireman does not have other objects beside his celery.

In contrast, in the LS condition, the focal meaning component is *I didn’t give celery to anyone else*. Verification of this proposition would require checking that the propositions making up the focal meaning component, i.e., (11c), are all true. This would mean looking at any people in the picture and their objects because these are potentially relevant entities for determining that the fireman is the only person who received a celery (see 11d). Since the utterance with late stress is actually false in Experiment 1, one of these propositions is false. The actual falsifying proposition in the visual context is ‘I gave celery to the diver’ (see 11e). In other words, it is due to the fact that the diver has a celery in the picture, that the utterance does not match the picture. For this reason, we expect that looks will target the diver and the diver’s celery. Without looking at these entities, it is simply impossible to reach the correct response. In addition, looks targeting the diver’s corn during the computation of the response in the LS condition are also consistent with our predictions but are not necessary to reach a correct response. While the diver’s corn is irrelevant for the focal meaning component, participants may look at it to verify that it is not celery. In addition, participants might target the doctor, to see if he has any celery. Since in our specific example the doctor’s plates are empty and the emptiness of a plate is easy to identify parafoveally, it is expected that the doctor’s empty plates are not directly targeted by looks.

To sum up, our hypothesis is that once participants proceed to verify the focal meaning component, we expect that looks will diverge across the two conditions. In particular, we predict that in the ES condition looks will stay on the fireman and the fireman’s celery, while in the LS condition, looks will shift to the diver, the diver’s celery and to a lesser extent to the diver’s corn (and perhaps even to the doctor). This could take place at the earliest during the indirect object or during the sentence final verb *gegeven* if semantic integration of prosodic focus is fast, and may occur after the utterance offset if semantic integration of prosodic focus is slower. If looks diverge in the predicted way already at the point of the indirect object, we would take that to be evidence for fast, incremental semantic integration of prosodic focus information.

There is one additional specific conclusion that we can draw, if our predictions are born out, irrespective of the fast or slow nature of the semantic integration. The proposed findings would constitute evidence that the verification process corresponds to the semantics associated with the utterance (see Horn, 1969; Krifka, 1992; Rooth, 1992 and discussion above). In principle, one may imagine that instead of looks corresponding directly to the focal meaning component, participants could engage in heuristic strategies. For argument’s sake, one may hypothesize for instance that in order to verify an utterance involving *only* it would be enough to look for the falsifying entity. So, in *I only gave celery to the FIREMAN* looks could target any celery in the picture that does not belong to the fireman. The participant could legitimately reject the utterance without having verified that this offending celery in fact belongs to the diver. In other words, looks to the falsifying entity are logically necessary for falsification, but looks to the possessor of that falsifying entity are not. If we find looks targeting the diver too, that would support the hypothesis that sentence verification follows the proposition-based semantics associated with *only*-sentences. In other words, we take looks to the diver (in addition to looks to the diver’s celery) as an indication that participants do not simply look for an offending object, but attempt to verify the relevant proposition of the focal meaning component, *I didn’t give celery to anyone else*, falsified by the proposition *I gave celery to the diver*. Naturally, this evidence is only indirect. We cannot be sure that looks to the diver necessarily mean that participants entertain the falsifying proposition. But the implication holds the other way: anyone entertaining the falsifying proposition would have to look at the diver as well as his celery.

## Experiment 1

### Materials and Methods

#### Participants

Twenty adult participants were recruited from the UiL OTS participant pool, which is largely made up of undergraduate students from Utrecht University. All participants were non-dyslectic native speakers of Dutch. Participants were unaware of the purpose of the experiment, and were paid 5€ for their participation. The mean age of the participants was 22;8 years (range: 19–29); 18 participants were females; 17 were right-handed. This study was carried out in accordance with research ethical laws of the Netherlands with informed consent from all subjects.

#### Materials

Sixteen items were constructed in two conditions. **Figure 1** above shows an example scene for both conditions. There are three persons in the picture; a diver, a doctor and a fireman in this example. The persons all hold large plates on their left and right side. Some of the plates are empty and some contain food or drink items, a celery or a corn cob in this example. In particular, the diver has a plate with a celery and one with a corn cob, while the fireman has a plate with a celery and an empty plate.

As shown by the example test items in (12) the expected response in the ES condition was YES, while it was NO in the LS condition. This allowed us to determine whether participants reached the correct response depending on the prosody of the item. This also allowed us to have results that are comparable to Gennari et al.’s (2005) study, although this difference does introduce a potential confound for response time measurements.

The visual scenes were designed using a 3 × 3 grid design, the distance between any two objects is identical and would be sufficiently large to be well-suited for eye-tracking evaluation. The pairs of objects in the pictures – corn and celery in **Figure 1** – were chosen to match in size, shape, and gray value, to make sure participants shift their gaze to them and not identify them parafoveally while looking at the person in the middle.

The audio stimuli corresponding to the visual scene in **Figure 1** are in (12). The full items list is in Appendix 3.

(12) a. ES condition: *Expected answer: YES*
  I have only celery to the fireman given
  ‘I only gave celery to the fireman.’
  b. LS condition: *Expected answer: NO*
    I have only celery to the fireman given
    ‘I only gave celery to the fireman.’

For practical reasons, the experiment was performed in Dutch. Dutch prosody is sufficiently similar to English prosody to allow comparison with previous work in English. Both languages mark contrastive focal accent by enhanced duration and H^∗^L pitch accent. Stress placement within an utterance is free to match the focus within the syntactic scope of the semantic operator. Relevant phonetic details for the examples in (12) are in Table S1 in Appendix 1.

**FIGURE 2:**
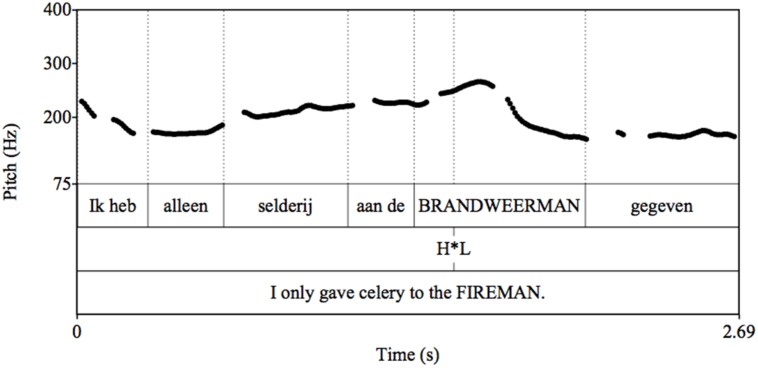
**Pitch track for example of Late Stress (LS) Condition utterance**.

**FIGURE 3:**
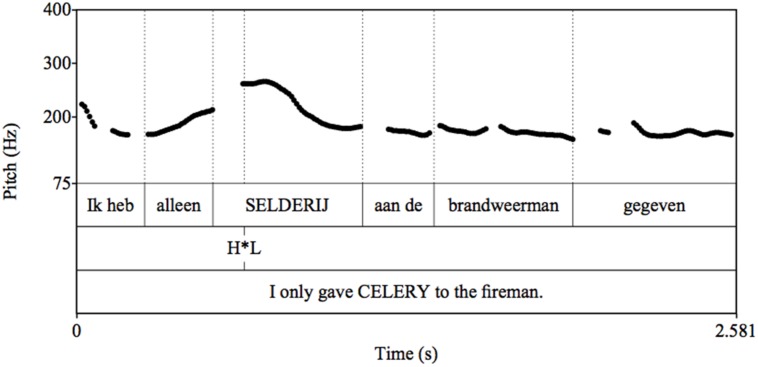
**Pitch track for example of Early Stress (ES) Condition utterance**.

Verbal stimuli were pre-recorded by a female native speaker of Dutch. They were checked for the placement of pitch accents using PRAAT (Boersma and Weenink, 2006); for examples see **Figures 2** and **3**.

The names of target objects and people that were used in the sentences were matched in length. They were all at least three syllables long. There were no significant differences between conditions in the overall lengths of the audio stimuli [*t*(16) = 0.95, *p* = 0.925].

Although the utterances as a whole do not differ in length in the two conditions, there are slight differences in length between conditions in certain auditory segments: the direct object segment was slightly longer when it was stressed (in the ES condition), as was the indirect object segment in the LS condition. These differences canceled each other out overall, since each condition contains exactly one segment with marked stress.

Ninety-eight filler items were constructed including various quantifiers (e.g., *niet iedereen* ‘not everybody’). The fillers were balanced for YES/NO expected responses. To match our test items, the fillers either involved early marked stress on the direct object or late marked stress on the indirect object. The fillers included a set of 32 control items involving *alleen*, 16 with early and 16 with late stress, where the expected response was different from the expected response of the corresponding test condition. This would discourage people from developing a strategy relating the position of the accent to the expected response in the test items (i.e., early stress = YES; late stress = NO). Finally, half of these control items referred to the ‘doctor’ (i.e., to the person in the middle in the visual stimulus), so that participants do not disregard the middle person in the picture in general. A list of the type of fillers used is in Table S2 in Appendix 1.

Furthermore, we controlled for potential confounds caused by the spatial location of objects in the picture by varying their positions on the top-down and the left-right axes. The falsifying entity for the LS condition (the diver’s celery in the example in **Figure 1**) appeared in four different positions: in four items it was located in the top left-hand corner of the picture (as in **Figure 1**); four times it appeared in the top right; four times in the bottom right; and four times in the bottom left.

#### Procedure

The participants were tested individually in a sound-treated booth. Prior to the experiment, they read an instruction sheet, which included the setting of the experiment (see Appendix 2 for a translation). This provided a context in which both utterances with early stress and utterances with late stress would be pragmatically felicitous. The participants’ task was to indicate whether the sentence matched the visual scene by pressing a button on a button box, using the dominant hand to give a YES response, and the non-dominant hand to give a NO response. This might have introduced a response time bias in favor of the ES condition.

The experiment was programmed in FEP (Veenker, 2005). Eye movements of the participants’ right eye were recorded with an EyeLink 1000 eye tracker in remote mode using a target sticker to track head movements, at a 500 Hz sampling rate. Participants were seated at a distance of 600–650 mm from the screen where the visual image was presented; the height of the participants’ chair was adjusted to get an optimal image of the eye.

After the experimenter had ensured a clear image of the pupil, corneal reflection, and target sticker, the experimenter left the participant booth and a 13-point calibration and validation procedure was initiated from the control room. These were repeated until the experimenter was satisfied that they were successful. Every stimulus was preceded by a fixation target in the middle of a blank screen. An automatic drift check was applied as the participant fixated this fixation target and a recalibration initiated if the drift check indicated a drift of more than 20 pixels. Participants were allowed 1000 ms to explore the visual scene before the utterance was presented. The whole procedure, including instruction and calibration, took about 20 minutes for each participant.

After successful calibration, the participants were exposed to a practice block of 12 practice items (fillers, 2 of those resembling experimental items), to familiarize them with the task. The practice block was followed by a small pause in which the participants could ask questions about the task (if necessary). After this, the experiment would start. The remaining 118 trials (32 test items, 32 controls, 54 fillers) were presented in two blocks; each block was preceded by a calibration.

All the names of the persons and objects involved in the experimental items were mentioned in the first 16 filler trials (including the practice block), to ensure that the participants had seen them and knew what they were called.

All participants saw all the test items in both conditions. The items and fillers were presented to the participants in a pseudo-randomized order where an experimental item never directly followed another experimental item in any condition; of the (experimental or filler) items involving *alleen* ‘only’, the trials with late stress never followed a trial with early stress or vice versa; and experimental items never followed a filler involving *alleen* ‘only’ with any stress pattern. No more than three trials with the same stress pattern occurred successively.

### Results

For the response data, two experimental trials belonging to the same participant were removed because the answer had already been given before onset of the indirect object. In addition, one filler trial was removed because the answer had been given before sentence onset.

#### Number of Correct Responses

The percentage of correct responses for the LS condition was 98%, for the ES condition 99%. The difference was not significant [*F*_1_(1,19) = 2.923, *p* = 0.104, $\eta_{p}^{2}$ = 0.133]. The overall correct response rate for the experiment was 98%.

#### Response Time

The overall mean response time from utterance onset for the LS condition was 3034 ms, while it was 3048 ms for the ES condition. The difference is not significant [*F*_1_(1,19) = 0.027, *p* = 0.871, $\eta_{p}^{2}$ = 0.001].

So, we did not find a significant effect in response time or accuracy across the conditions.

#### Eye Gaze Patterns

##### Coding and analysis

We identified six Areas of Interest, the ‘fireman’, the ‘fireman’s celery’, the ‘diver’, the ‘diver’s celery’, the ‘diver’s corn,’ and the ‘doctor’. See **Figure 1**. Fixations were assigned to the AoI they occurred on. For ease of reference, we refer to the AoIs with the content of the example stimulus in **Figure 1**; the data and plots that we give are calculated over all items and participants, so when for instance we say ‘fireman’, we mean ‘the person in the picture who is part of the non-focal proposition in all the items’.

For the fine-grained analysis of eye movements over time, we divided the utterances into relevant audio segments, and determined the onset of each segment using PRAAT. The sentence segments are: *selderij/SELDERIJ* ‘celery/CELERY’; *aan de* ‘to the’; *BRANDWEERMAN/brandweerman* ‘FIREMAN/fireman’; *gegeven* ‘given’. For each segment, we analyzed the fixation samples falling between 200 ms after segment onset, and 200 ms after the offset of that auditory segment, to take into account that it takes 200 ms to launch a saccade driven by linguistic input (cf. Altmann and Kamide, 2004). The final three segments comprise the interval starting at 200 ms after the onset of the verb *gegeven* ‘given’ and ending 1500 ms later. This was divided into three segments of identical length. For ease of reference we call these the auditory segment *gegeven* ‘given’, the ‘first 500 ms interval after offset’ and the ‘second 500 ms interval after offset’. For reference, the average durations of the utterance components are given in Table S3 in Appendix 1.

For each experimental trial, we computed the proportion of time the participant spent fixating each area of interest in each auditory segment. We averaged these proportions over participants for the ES and LS conditions. We carried out six two-way repeated measures ANOVAs (using SPSS 22), one for each auditory segment, using Visual AoI and Condition as the two main factors. The results are reported in **Table 1**. We also provide effect size measures ($\eta_{p}^{2}$).

**Table 1:** Analysis of variance per auditory segment for Experiment 1.

Auditory segment	Factor	df1	df2	*F*_1_	*p*	$\eta_{p}^{2}$
Direct object	AoI	3.689^a^	70.087^a^	4.814	0.001^∗^	0.202
	Condition	1	19	4.682	0.043^∗^	0.198
	AoI^∗^condition	5	95	2.113	0.071^†^	0.100
*aan de* ‘to the’	AoI	3.415^a^	64.885^a^	24.715	0.000^∗^	0.565
	Condition	1	19	0.223	0.642	0.012
	**AoI^∗^condition**	**3.729^a^**	**70.849^a^**	** 2.747**	**0.023^∗^**	**0.126**
Indirect object	AoI	4.148^a^	78.805^a^	104.798	0.000^∗^	0.847
	Condition	1	19	5.586	0.029^∗^	0.227
	**AoI^∗^condition**	**4.032^a^**	**76.607^a^**	**8.793**	**0.000^∗^**	**0.316**
*gegeven* ‘given’	AoI	3.015^a^	57.278^a^	65.559	0.000^∗^	0.775
	Condition	1	19	0.950	0.342	0.048
	**AoI^∗^condition**	**5**	**95**	** 28.532**	**0.000^∗^**	**0.600**
0–500 ms after offset	AoI	3.064^a^	58.217^a^	30.372	0.000^∗^	0.615
	Condition	1	19	1.438	0.245	0.070
	**AoI^∗^condition**	**2.210^a^**	**41.998^a^**	** 19.112**	**0.000^∗^**	**0.501**
501–1000 ms after offset	AoI	2.412^a^	45.828^a^	16.588	0.000^∗^	0.466
	Condition	1	19	0.395	0.537	0.020
	**AoI^∗^condition**	**2.884^a^**	**54.803^a^**	** 3.907**	**0.003^∗^**	**0.171**


*^a^Huynh-Feldt corrected.*

We found a significant main effect for AoI in all auditory segments, meaning that participants look more to some AoIs than to others in all auditory segments. We also found a significant main effect for Condition in the direct object and the indirect object auditory segments, which means that one condition has a more unequal distribution of the proportion of looks than the other. Since they are not relevant to our research question, we do not investigate these main effects further. Although these may well contain interesting information about how our test sentences are processed, in this paper, we concentrate on the differences in eye gaze patterns that can be attributed to the prosodic difference across the conditions, which is manifested in the interactions between Visual AoI and Condition. This is consistent with our intention to investigate whether (and if so when) there is any effect of the position of prosodic focus (i.e., early versus late) on the eye gaze patterns associated with auditory sentence processing. Thus, the most relevant findings are that we found significant interactions of Visual AoI and Condition for the *aan de* ‘to the’, the indirect object, the verb (*gegeven* ‘given’) time segments, and for the first and second 500 ms interval after utterance offset. These are indicated in bold in **Table 1** for ease of reference. For these auditory segments, we carried out pairwise comparisons, applying Bonferroni corrections, to reveal which AoIs were targetted by eye fixations differently, at any segment, across the two conditions. The significant results are reported in **Table 2**. Statistically significant differences are indicated with an asterisk, and marginally significant effects are marked with a dagger throughout the paper.

**Table 2:** Proportions of time spent fixating each of the relevant people and objects in Early Stress (ES) and Late Stress (LS) condition for each auditory segment, and pairwise comparisons between conditions with Bonferroni correction applied for Experiment 1.

Auditory segment	Person/object	Proportion of time looking in ES	Proportion of time looking in LS	*F*_1_(1,19)	*p*	$\eta_{p}^{2}$
Indirect object	Diver	0.10	0.14	10.557	0.004^∗^	0.357
	Diver’s celery	0.04	0.10	15.540	0.001^∗^	0.450
	Fireman	0.42	0.33	14.756	0.001^∗^	0.437
*gegeven* ‘given’	Diver	0.05	0.14	29.426	0.000^∗^	0.608
	Diver’s celery	0.04	0.12	13.907	0.001^∗^	0.423
	Fireman	0.42	0.24	83.212	0.000^∗^	0.814
	Fireman’s celery	0.19	0.12	9.824	0.005^∗^	0.341
0–500 ms after offset	Diver	0.04	0.10	14.356	0.001^∗^	0.430
	Diver’s celery	0.02	0.08	25.298	0.000^∗^	0.571
	Diver’s corn	0.01	0.04	8.803	0.008^†^	0.317
	Fireman	0.29	0.13	20.688	0.000^∗^	0.521
	Fireman’s celery	0.13	0.08	6.946	0.016^†^	0.268


**FIGURE 4:**
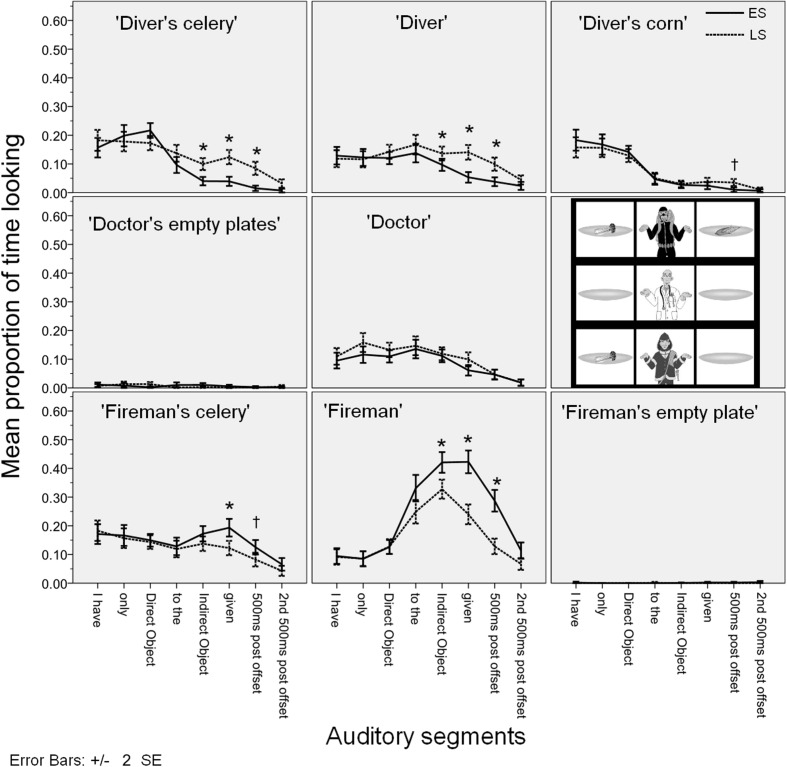
**Time course of the mean fixation time proportions for each AoI in Experiment 1**.

**Figure 4** gives the time course of the mean fixation time proportions for each AoI in each auditory segment in Experiment 1. The figure contains 8 plots corresponding to the nine cells in our 3 × 3 visual scene (the ‘doctor’s empty plates’ are plotted together). In each plot we give the mean proportion of time spent looking at that cell (e.g., the ‘fireman’ etc.) during each auditory segment in the two conditions (ES vs. LS). The error bars indicate ±2 SE.

### Discussion

#### Behavioural Measures

We found a high number of correct responses in both conditions and no significant difference in the number of correct judgments between the conditions. We speculate that the reason why we got a higher number of correct responses than Gennari et al. (2005) may have been because their experiment involved a more realistic and more complex visual scene, while ours was a stylised 3 × 3 design, and because we provided an overall context story, while participants in the Gennari et al. (2005) study heard items without context.

Like Gennari et al. (2005), we did not find any significant differences in response times between the conditions. Importantly, this result may have been influenced by the difference in expected responses (ES: YES, LS: NO), as in the original Gennari et al. (2005) study. It is possible that it takes longer (or shorter) to find a falsifying entity in a picture than it takes to scan the picture and verify that there is no falsifying entity present. It could also be the case that a negative judgment takes longer (or shorter) than a positive one. Moreover, it is possible that the use of the non-dominant hand to tap a NO response introduced a bias in favor of the ES condition. In order to control for these factors, we performed Experiment 2, where the expected response in both conditions was YES. In Experiment 3, the expected response was NO in both conditions.

#### Eye Gaze Patterns

The most important finding from Experiment 1 is that looks started diverging across conditions in the predicted way during the indirect object time segment: More looks targeted the ‘diver’ and the ‘diver’s celery’ in the LS condition than in the ES condition and more looks targeted the ‘fireman’ in the ES condition than in the LS condition. This provides evidence that participants have computed the focal meaning component as early as the indirect object time segment: so focus information was integrated into the semantic parse of the utterance very fast. This is because the observed divergent looks correspond to the participants’ attempt at verifying the focal meaning component of the utterances, which is different in the two conditions [see (10c) and (11c) above].

The looks follow the predictions further at the sentence-final verb *gegeven* ‘given’ and after the utterance offset: participants’ looks target the ‘fireman’ and the ‘fireman’s celery’ more in the ES condition, while looks target the ‘diver’, the ‘diver’s celery’, and somewhat later also the ‘diver’s corn’ in the LS condition (the effect on the ‘diver’s corn’ is right at the Bonferroni-corrected alpha level, to be on the conservative side we take it to be marginally significant). As expected, the effect was longer on the ‘diver’s celery’ and the ‘diver’ as these correspond to the actual falsifying proposition in the LS condition, and it was shorter and less pronounced on the ‘diver’s corn’ which is an entity that is only *potentially* relevant for falsification.

These findings also constitute evidence that the verification process corresponds to the semantics associated with the utterance (see Horn, 1969; Krifka, 1992; Rooth, 1992 and discussion above). Participants do not simply look for an offending object (i.e., ‘diver’s celery’), their looks also target the person holding that object (i.e., ‘diver’). We take this to mean that they are verifying the relevant proposition of the focal meaning component, *I didn’t give celery to anyone else*, falsified by the proposition *I gave celery to the diver.*

Our expectation that looks follow the logic of the semantically determined focal meaning component is further supported by the relative absence of looks to irrelevant entities. While participants do look at the (potentially) falsifying entities (the ‘fireman’s celery’ in the ES condition; the ‘diver’s celery’ in the LS condition), they do not target Gennari et al.’s (2005) ‘contrast entities’, i.e., the ‘diver’s corn’ in the ES condition. At no auditory segment are looking proportions to the ‘diver’s corn’ higher in the ES condition than in the LS condition.

Overall, our findings are consistent with early and incremental focus identification and association with *only*, consolidating earlier results by Gennari et al. (2005), Paterson et al. (2007), and Ito and Speer (2008), and pinpointing the effect to the earliest point in time that participants have the necessary information to compute the meaning components of the utterance, namely the indirect object. No facilitation was found for response times. However, this may have been due to the fact that the two conditions had divergent expected responses in Experiment 1. We investigate this issue further in Experiment 2.

As a final point, recall that our research aim is to determine the earliest point at which participants’ looks give evidence of distinguishing the two conditions. In general, in visual-world eye-tracking, we can observe that fixation proportions on a particular AoI always grow gradually, eventually reaching a peak, and then gradually diminishing. As a result, any robust difference we find in a particular sound segment is likely to be immediately preceded (and followed) by a less robust difference in the preceding (or following) sound segment. As noted above, we found a predicted difference between time spent fixating on the ‘fireman’ in the ES and the LS conditions, which is significant (with Bonferroni correction) from the indirect object segment onwards. But as **Figure 4** shows, the difference in looks to the ‘fireman’ across the conditions seems to start to grow already during the *aan de* ‘to the’ segment. At this segment, the effect is not robust under correction for multiple comparisons [*F*_1_(1,19) = 6.006, *p* = 0.024, $\eta_{p}^{2}$ = 0.240], so we cannot draw any strong conclusions from it, but it is an indication that the effect may start to happen earlier than we expected. This is surprising because it suggests that participants start looking at the ‘fireman’ before they have actually heard the noun *brandweerman* ‘fireman’. We interpret the potential early start of the effect as the participants’ anticipating the continuation of the utterance to be *brandweerman* ‘fireman’ at the point when they have heard *Ik heb alleen SELDERIJ aan de…* ‘I only (gave) CELERY to the…’. We tested this hypothesis in Experiment 3.

## Experiment 2

### Material and Methods

#### Participants

Twenty non-dyslexic native Dutch speakers were recruited from the UiL OTS participant pool. Participants were unaware of the purpose of the experiment, and were paid 5€ for their participation. The mean age of the participants was 24;3 years (range: 19–46); 19 females and 1 male; 17 participants were right-handed.

#### Materials

**FIGURE 5:**
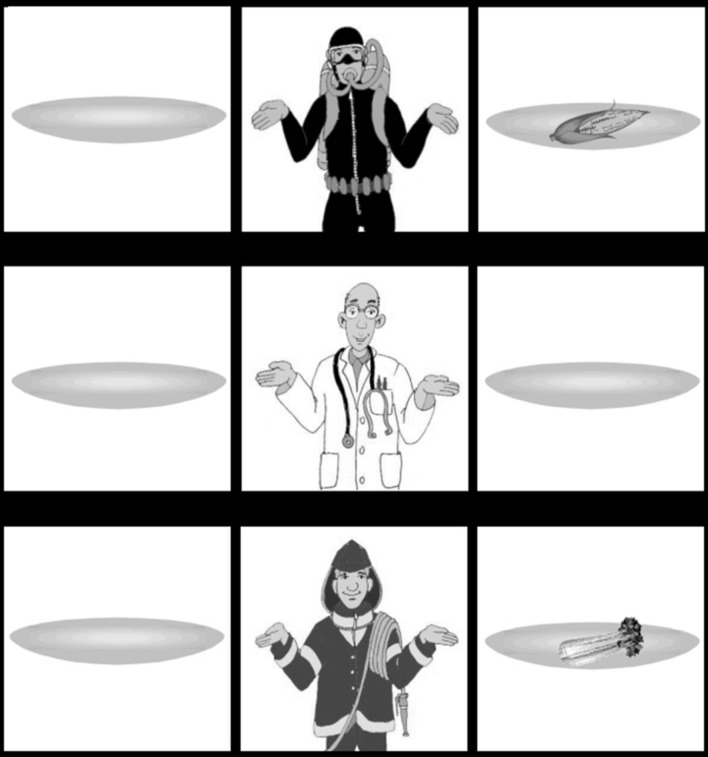
**Example of visual stimulus for Experiment 2**.

Like in Experiment 1, 16 items were constructed in two conditions. The auditory stimuli were identical to the ones used in Experiment 1. The visual stimuli of Experiment 1 were changed in such a way that the expected responses were YES in both conditions. In particular, the ‘diver’ had a plate with a ‘corn cob’ and an empty plate, while the ‘fireman’ had a plate with a ‘celery’ and an empty plate. See **Figure 5** for an example.

The 64 unrelated fillers from Experiment 1 were included alongside the test items. In addition 32 controls involving *alleen* ‘only’ were created, 16 with early stress, 16 with late stress, with half of the items referring to the ‘doctor’. The expected response for the controls was NO, to counterbalance the YES bias introduced by the test items.

#### Procedure

The procedure was identical to that of Experiment 1.

### Predictions

We were interested to see if there was a facilitatory effect of early stress resulting in shorter response times in the ES condition compared to the LS condition. Regarding eye movement patterns, our predictions were the same as in Experiment 1 except that since both conditions are true in the pictures, there is no falsifying entity in the picture.

### Results

Two trials from different experimental participants were removed from the response data because the response was given before the indirect object segment.

#### Number of Correct Responses

The percentage of correct responses for the LS condition was 100%, for the ES condition 99%. The difference was not significant [*F*_1_(1,19) = 0.322, *p* = 0.577, $\eta_{p}^{2}$ = 0.017]. The overall correct response rate for the experiment as a whole was 98%.

#### Response Time

The overall mean response time from utterance onset for the LS condition was 2843ms, while it was 2868 ms for the ES condition. The difference is not significant [*F*_1_(1,19) = 0.147, *p* = 0.706, $\eta_{p}^{2}$ = 0.008].

#### Eye Gaze Patterns

*Coding and analysis* was identical to that in Experiment 1.

**FIGURE 6:**
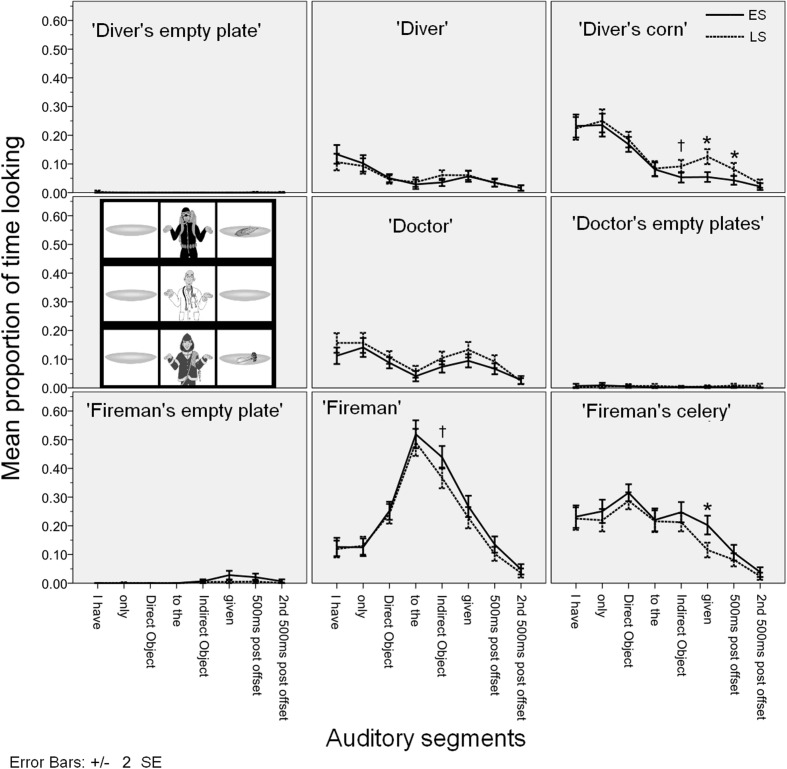
**Time course of the mean fixation time proportions for each AoI in Experiment 2**.

**Figure 6** gives the mean proportion of looking time for each AoI in the auditory segments.

**Table 3** gives the analysis of variance for Experiment 2.

**Table 3:** Analysis of variance per auditory segment for Experiment 2.

Auditory segment	Factor	df1	df2	*F*_1_	*p*	$\eta_{p}^{2}$
Direct object	AoI	2.351^a^	44.671^a^	32.373	0.000^∗^	0.630
	Condition	1	19	0.914	0.351	0.046
	AoI^∗^condition	2.869^a^	54.514^a^	0.952	0.419	0.048
*aan de* ‘to the’	AoI	1.874^a^	35.606^a^	108.419	0.000^∗^	0.851
	Condition	1	19	0.215	0.648	0.011
	AoI^∗^condition	2.571^a^	48.857^a^	0.551	0.623	0.028
Indirect object	AoI	2.468^a^	46.885^a^	72.450	0.000^∗^	0.792
	Condition	1	19	1.061	0.316	0.053
	**AoI^∗^condition**	**3.339^a^**	**63.437^a^**	** 5.486**	**0.001^∗^**	**0.224**
*gegeven* ‘given’	AoI	2.089^a^	39.684^a^	18.029	0.000^∗^	0.487
	Condition	1	19	0.213	0.649	0.011
	**AoI^∗^condition**	**4**	**76**	** 9.312**	**0.000^∗^**	**0.329**
0–500 ms after offset	AoI	3.050^a^	57.953^a^	8.651	0.000^∗^	0.313
	Condition	1	19	0.014	0.907	0.001
	**AoI^∗^condition**	**3.647^a^**	**69.295^a^**	** 3.946**	**0.008^∗^**	**0.172**
501–1000 ms after offset	AoI	2.740^a^	52.055^a^	2.690	0.061	0.124
	Condition	1	19	0.572	0.459	0.029
	AoI^∗^condition	2.932^a^	55.700^a^	1.064	0.371	0.053


*^a^Huynh-Feldt corrected.*

Like in Experiment 1, we were interested in the interaction between Visual AoI and Condition, because this would reveal any potential effect of the different positioning of focal accent on sentence processing. The interaction between AoI and Condition was significant during the indirect object, the verb *gegeven* ‘given’, and during the first 500 ms after the verb. These are indicated with bold in **Table 3** for ease of reference. To reveal where the actual differences in eye gaze patterns across the conditions lied, we performed pairwise comparisons for these auditory segments. The significant Bonferroni-corrected results are given in **Table 4**.

**Table 4:** Proportions of time spent fixating each of the relevant people and objects in ES and LS condition for each auditory segment, and pairwise comparisons between conditions with Bonferroni corrections applied for Experiment 2.

Auditory segment	Person/object	Proportion of time looking in ES	Proportion of time looking in LS	*F*_1_(1,19)	*p*	$\eta_{p}^{2}$
Indirect object	Diver’s corn	0.05	0.09	6.777	0.017^†^	0.263
	Fireman	0.44	0.37	8.070	0.010^†^	0.298
*gegeven* ‘given’	Diver’s corn	0.05	0.13	17.702	0.000^∗^	0.482
	Fireman’s celery	0.20	0.12	22.323	0.000^∗^	0.540
0–500 ms after offset	Diver’s corn	0.04	0.08	9.169	0.007^∗^	0.326


Looks started diverging across conditions during the indirect object auditory segment, where more time was spent looking at the ‘diver’s corn’ in the LS condition, and more time was spent looking at the ‘fireman’ in the ES condition. On the ‘diver’s corn’ the effect continued during the auditory segments corresponding to the verb, and the first 500 ms intervals after utterance offset. During the utterance final verb *gegeven* ‘given’, there was also significantly more time spent targeting the ‘fireman’s celery’ in the ES condition.

### Discussion

#### Behavioral Measures

We found a high rate of correct responses for both test conditions. We did not find that the early occurrence of stress facilitated verification, as response times did not differ across conditions. See Section “General Discussion” below for more on this.

#### Eye Gaze Patterns

The eye gaze patterns were similar to Experiment 1. The looking patterns diverged in the expected way: more looks on the ‘fireman’ and the ‘fireman’s celery’ in the ES condition and on the ‘diver’s corn’ in the LS condition. Perhaps due to the more simple nature of the visual stimulus, the effects are not as sustained over time as they are in Experiment 1.

Let us now turn to our final experiment, where the expected responses were NO in both conditions.

## Experiment 3

### Introduction

Recall that in Experiment 1, we found that the difference between conditions in the looks targeting the ‘fireman’ seems to start already before the indirect object was heard. Although we did expect more looks targeting the ‘fireman’ in the ES condition, we did not expect this to happen until after the indirect object *de brandweerman* ‘the fireman’ was actually heard. We believe that this increase in looks targeting the ‘fireman’ in the ES condition is anticipatory. Our hypothesis is that in a picture verification task, participants employ an unconscious strategy when performing the task: they start out with the assumption that the utterance will match the picture.

Let us explain this in more detail using our actual example. Take the moment when participants hear the first half of the utterance *Ik heb alleen SELDERIJ …* ‘I have only CELERY …’ in a setting where the fireman is the only person that has only celery, as in **Figure 1** from Experiment 1. At this point, there is only one continuation of this utterance that would make the sentence true in the picture, namely the actual continuation (i.e., … *aan de brandweerman gegeven.* ‘…to the fireman given.’). Any alternative continuation (e.g., referring to the ‘diver’) would make the sentence false. Given that there is only one way a sentence can be true in a picture and there are many ways it could be false, it would make sense for the listener to adopt a cognitive strategy that assumes that the sentence is true until proven wrong. In contrast, in the LS condition, when participants hear *Ik heb alleen selderij …* ‘I have only celery …’ in a context of a picture where both the fireman and the diver has celery, as in **Figure 1**, there is no continuation of the utterance that can make the sentence true. So, there is no anticipatory advantage in this condition. This has the effect that there is a stronger tendency for participants’ eye gaze to already target the ‘fireman’ in the ES condition than in the LS condition, even before they have heard the word *brandweerman* ‘fireman’ (i.e., during the *aan de* ‘to the’ auditory segment).^6^ In short, we speculate that in a bi-modal verification task, participants anticipate the continuation of the sentence to be such that it makes the utterance true in the picture.

This is in line with findings of Altmann and Kamide (1999) and Kamide et al. (2005) (see also Ito and Speer, 2008). These authors found that while listening to utterances presented to them in the visual-world paradigm, participants can sometimes anticipate certain semantic properties of forthcoming lexical items based on the lexical items they have already heard. In particular, they tested utterances like *The boy will eat the cake* presented in a visual setting with a boy, a cake, a toy car, a toy train and a ball. They found that participants’ eye movements targeted the cake already before the object noun phrase, so when they only heard *The boy will eat….* Altmann and Kamide (1999) showed that this was because the verb *eat* places a semantic restriction on the object noun phrase and the cake was the only edible object in the picture.

The goal of Experiment 3 was to investigate our anticipatory look hypothesis.

### Material and Methods

#### Participants

Twenty-three non-dyslexic native speakers of Dutch were recruited from the UiL OTS participant pool.

Data of three participants were discarded prior to analysis; one participant did not receive adequate instruction prior to the experiment and reread the instruction sheet repeatedly during the experiment; one experimental run suffered from an unresponsive button box; and one participant was intimately familiar with linguistic theories on stress shift. The remaining participants were unaware of the purpose of the experiment, and were paid 5€ for their participation. The mean age of the 20 remaining participants was 22;8 years, ranging from 17 to 27 years; 14 females and 6 males; 18 participants were right-handed.

#### Materials

**FIGURE 7:**
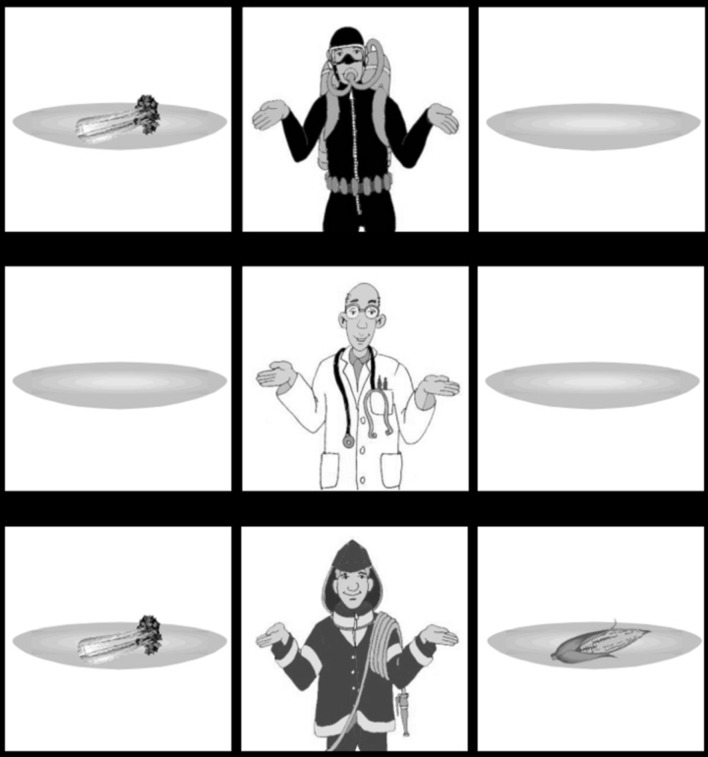
**Example of visual stimulus for Experiment 3**.

Like in Experiments 1 and 2, 16 items were constructed in two conditions. The auditory stimuli were identical to the ones used in Experiments 1 and 2. The visual stimuli were changed in such a way that the expected responses were NO in both conditions. In particular, the ‘fireman’ had a ‘celery’ and a ‘corn cob’, while the ‘diver’ had a ‘celery’. See **Figure 7** for an example.

The 64 unrelated fillers from Experiments 1 and 2 were included alongside the test items. In addition 32 controls were created, 16 with early stress, 16 with late stress; half of the items mentioning the ‘doctor’. The expected response for the controls was YES, to counterbalance the test items.

#### Procedure

The procedure was identical to that of Experiments 1 and 2.

### Predictions

The experiment was designed to test our hypothesis that the trend toward an early increase in looks targeting the ‘fireman’ in Experiment 1 was anticipatory in the following sense. Participants hear *Ik heb alleen SELDERIJ…* ‘I have only CELERY…’ and anticipate that the utterance will continue in such a way that it matches the picture. In Experiment 3 the visual stimulus was changed in such a way that the only person that has only celery is the ‘diver’. See **Figure 7**. The audio stimuli were identical to that of Experiment 1. Thus, our anticipation hypothesis predicts that participants will look more at ‘the diver’ during the *aan de* ‘to the’ auditory segment in the ES condition. This is because the ‘diver’ has only ‘celery’. But once they hear the indirect object *de brandweerman* ‘the fireman’, their looks are expected to shift to the ‘fireman’. So, it is expected that in Experiment 3 the anticipatory strategy ‘tricks’ participants.

In addition, we expected that the findings of Experiments 1 and 2 about divergent looks between the two conditions would be replicated, except potentially, due to the potential hindering effect of the anticipatory looks, somewhat delayed.

### Results

Eleven trials (six experimental) were removed from analysis of the response data because the response had already been given before the onset of the indirect object (six experimental and four filler items) or utterance onset (one filler).

#### Number of Correct Responses

The percentage of correct responses for the LS condition was 97%, for the ES condition 98%. The difference was not significant [*F*_1_(1,19) = 1.353, *p* = 0.259, $\eta_{p}^{2}$ = 0.066]. The overall correct response rate for Experiment 3 was 97%.

#### Response Time

The overall mean response time from utterance onset for the LS condition was 2875 ms, while it was 2909 ms for the ES condition. The difference is not significant [*F*_1_(1,19) = 0.418, *p* = 0.526 $\eta_{p}^{2}$ = 0.022].

#### Eye Gaze Patterns

*Coding and analysis* was identical to that in Experiments 1 and 2.

**Table 5** gives the analysis of variance for Experiment 3. Like in Experiments 1 and 2, we focus only on the significant interactions between Visual AoI and Condition, as those are the relevant findings for our research question. For ease of reference, these are given in bold.

**Table 5:** Analysis of variance per auditory segment for Experiment 3.

Auditory segment	Factor	df1	df2	*F*_1_	*p*	$\eta_{p}^{2}$
Direct object	AoI	5	95	7.429	0.000^∗^	0.281
	Condition	1	19	0.105	0.750	0.005
	AoI^∗^condition	5	95	1.273	0.282	0.063
*aan de* ‘to the’	AoI	5	95	8.342	0.000^∗^	0.305
	Condition	1	19	3.147	0.092	0.142
	**AoI^∗^condition**	**5**	**95**	**3.434**	**0.007^∗^**	**0.153**
Indirect object	AoI	3.348^a^	63.620^a^	34.894	0.000^∗^	0.647
	Condition	1	19	5.637	0.028^∗^	0.229
	**AoI^∗^condition**	**5**	**95**	**3.894**	**0.003^∗^**	**0.170**
*gegeven* ‘given’	AoI	5	95	43.086	0.000^∗^	0.694
	Condition	1	19	20.789	0.000^∗^	0.522
	**AoI^∗^condition**	**5**	**95**	**16.929**	**0.000^∗^**	**0.471**
0–500 ms after offset	AoI	3.856^a^	73.263^a^	16.362	0.000^∗^	0.463
	Condition	1	19	12.266	0.002^∗^	0.392
	**AoI^∗^condition**	**4.178^a^**	**79.389^a^**	**11.201**	**0.000^∗^**	**0.371**
501–1000 ms after offset	AoI	2.652^a^	50.383^a^	6.628	0.000^∗^	0.259
	Condition	1	19	0.152	0.701	0.008
	**AoI^∗^condition**	**3.310^a^**	**62.893^a^**	**5.869**	**0.000^∗^**	**0.236**


*^a^Huynh-Feldt corrected.*

We find a significant interaction between AoI and Condition in the *aan de* ‘to the’ auditory segment, during the indirect object, during the verb *gegeven* ‘given’, and in the two auditory segments after utterance offset. Like in Experiments 1 and 2, the significant Bonferroni-corrected pairwise comparisons for these auditory segments are given in **Table 6**.

**Table 6:** Proportions of time spent fixating each of the relevant people and objects in ES and LS condition for each auditory segment, and pairwise comparisons between conditions with Bonferroni corrections applied for Experiment 3.

Auditory segment	Person/object	Proportion of time looking in ES	Proportion of time looking in LS	*F*_1_ (1,19)	*p*	$\eta_{p}^{2}$
*aan de* ‘to the’	Diver	0.27	0.18	7.518	0.013^†^	0.284
Indirect object	Fireman’s celery	0.07	0.11	8.288	0.010^†^	0.304
*gegeven* ‘given’	Diver’s celery	0.04	0.11	15.733	0.001^∗^	0.453
	Fireman	0.33	0.20	28.690	0.000^∗^	0.602
	Fireman’s corn	0.18	0.09	21.543	0.000^∗^	0.531
0–500 ms after offset	Diver’s celery	0.02	0.07	19.961	0.000^∗^	0.512
	Fireman	0.15	0.07	13.266	0.002^∗^	0.411
	Fireman’s corn	0.13	0.06	16.140	0.001^∗^	0.459
501–1000 ms after offset	Diver’s celery	0.01	0.04	14.628	0.001^∗^	0.435


**FIGURE 8:**
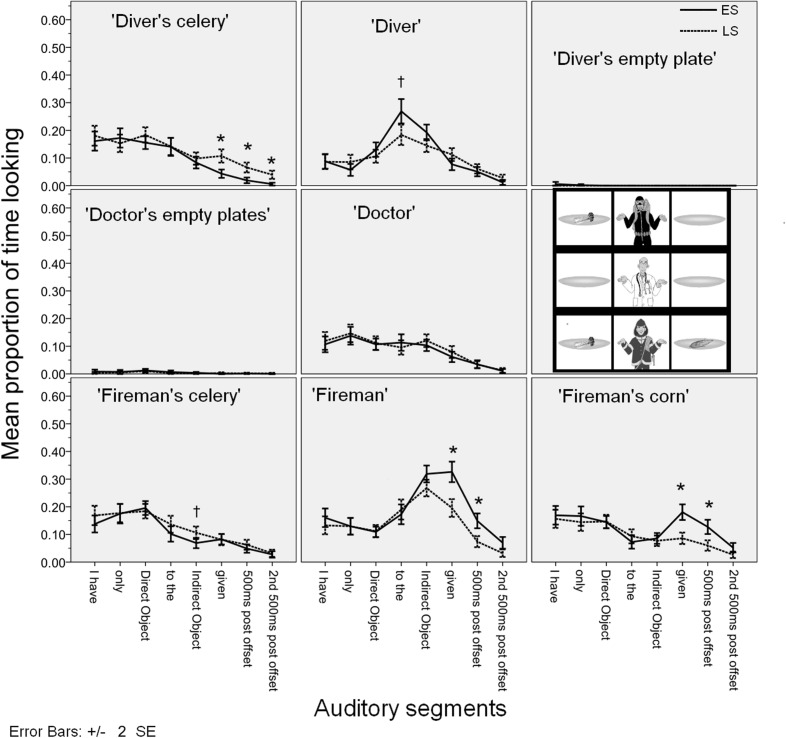
**Time course of the mean fixation time proportions for each AoI in Experiment 3**.

**Figure 8** gives the mean proportion of looks for each AoI in each auditory segment.

### Discussion of Experiment 3 and General Discussion

#### Behavioral Measures

Overall, in none of the experiments was there any facilitatory effect of the early occurrence of stress in terms of shorter response times or a higher accuracy rate for the ES condition. We think that this is because even though participants may use the earliness of stress to anticipate the continuation of the utterance, they still have to wait until the sentence is actually finished until they can establish the meaning components based on the actual continuation. So, overall, even if accentual information is presented earlier in the ES condition, leading to early identification of focus, this cannot facilitate computation of the meaning components associated with *only* overall, due to the propositional nature of these meaning components.

#### Eye Tracking Patterns

The visual stimulus in Experiment 3 was designed to test our hypothesis that participants anticipate the continuation of the utterance when they hear *Ik heb alleen SELDERIJ aan de…* ‘I have only CELERY to the…’ in the ES condition. If the sentence would continue with *duiker* ‘diver’, it would match the picture; and in the ES condition we do indeed find marginally more time is being spent looking at the ‘diver’ than in the LS condition, right before the indirect object is heard. Given that this effect is the start of a fixation curve (i.e., gradual growth, followed by robust peak, followed by gradual diminishing effect), we did not expect a more robust effect at this point. So, we can confirm our anticipation hypothesis.

But, the actual continuation of the utterance turns out to be *brandweerman* ‘fireman’. So, the utterance ends up being false. As predicted (and already found in Experiments 1 and 2), once the indirect object has been heard, looks shift to the ‘fireman’ and his possessions in the ES condition and to the ‘diver’s celery’ in the LS condition. The effects are somewhat delayed compared to Experiment 1, presumably due to the hindering effect of the anticipation strategy. During the sentence final verb, there are more looks targeting the ‘fireman’ and the ‘fireman’s corn’ in the ES condition and more looks targeting the ‘diver’s celery’ in the LS condition. We also expected that at this point, looks to the ‘diver’ would be higher in the LS condition than in the ES condition,— the direct opposite of our expectation for the *aan de* ‘to the’ auditory segment. Looks to the ‘diver’ are indeed numerically higher in the LS condition during the verb *gegeven* ‘given’ but the effect does not reach significance [*F*_1_(1,19) = 6.576, *p* = 0.019, $\eta_{p}^{2}$ = 0.257]. The expected effects on the ‘fireman’, the ‘fireman’s corn’ and the ‘diver’s celery’ continue throughout the first 500 ms interval after utterance offset. For the ‘diver’s celery’, the effect is still present during the second 500 ms after utterance offset. In addition, a marginally higher proportion of looks targetted the ‘fireman’s celery’ in the LS condition during the indirect object auditory segment, which was unexpected. We interpret this as the participants verifying the non-focal meaning component (i.e., that the ‘fireman’ has ‘celery’), which perhaps does not occur in the ES condition at this point due to the hindering effect of the mis-anticipated continuation.

Overall, we found that sentence verification starts early. In fact, perhaps unexpectedly, it starts already before the whole utterance is heard. Participants anticipate the continuation of the utterance assuming that the utterance will turn out to match the picture. Crucially, prosodic focus on the direct object was found to be relevant for guiding anticipatory looks already at the next sound segment, during *aan de* ‘to the’ (see Experiment 3). This gives evidence of incremental prosodic focus processing.

In all three experiments, we found that utterance verification proceeds according to the semantics of *only-*sentences (Horn, 1969; Krifka, 1992; Rooth, 1992). Participants’ looks robustly diverge already during the sentence final verb *gegeven* ‘given’ in the two conditions in all three experiments, as they verify the different focal meaning components associated with early and late occurrence of stress. In Experiment 1, robust effects were already found during the indirect object. So, we found evidence that participants’ looks not only target the falsifying entity in the picture, but rather the falsifying proposition was established. This provides support for the psychological reality of proposition-based semantics for prosodic focus association with *only*.

## Conclusion

Our results show incremental focus processing and thus fall in line with earlier results (Gennari et al., 2005; Paterson et al., 2007). Investigating the time course of looks accompanying the computation of *only-*sentences allowed us to pinpoint the time course of the semantic processing associated with focal differences that are marked prosodically. We found that people process prosodic focus immediately: there is evidence of participants verifying the focal meaning component already during the indirect object. We also found that participants make anticipatory looks in this picture verification task taking into account the prosodic focus of the utterance, providing further evidence of incremental focus computation at the earliest possible point, at the point where the direct object with or without prosodic focus is heard.

## Author Contributions

KS is responsible for the original design. IM determined the procedure and was responsible for creating the materials, and implementing and carrying out the experiment. IM carried out the data analysis and statistical calculations. KS and IM interpreted the data together and co-wrote the discussions.

## Conflict of Interest Statement

The authors declare that the research was conducted in the absence of any commercial or financial relationships that could be construed as a potential conflict of interest.

The reviewer BF and handling Editor declared a current collaboration and the handling Editor states that the process nevertheless met the standards of a fair and objective review.

## Footnotes

1 For ease of exposition, we illustrate the characteristics of focal stress and *only* with English examples. Everything we claim here holds for Dutch in the same way.

2 Note that (4a) with indirect object stress is in fact ambiguous in itself between the readings indicated in (i) and (ii). This is not important for the present study for two reasons. First, adults strongly prefer the reading in (i), which is the one targeted in this study (Crain and Steedman, 1985). Second, the experiments involve phonetically marked accent on the indirect object, which again makes the reading in (i) to be the preferred one. (i) The only person I gave some tea to was the woman = indirect object focus reading (ii) The only thing I did was give some tea to the woman = verb phrase focus reading

3 *Only* cannot associate with just any focus-bearing element; the focus-bearing element must be in its scope syntactically. Utterances like (3) are syntactically distinct from utterances like (i) in the sense that *only* in (3) is a verb-phrase-level adverb, while it directly modifies the subject noun phrase in (i). (i) Only [the WOMAN] gave a banana to the monkey. The kind of ambiguity that was present in (3) with *only* modifying the verb phrase, disappears in (i), because the operator *only* takes scope over its c-command domain, which is the verb phrase in (3) and the subject noun phrase in (i). Therefore, (i) can only have the reading exemplified in (iia). (ii) a. The only person that gave a banana to the monkey was the woman. b. ^∗^The only event that took place was the woman giving a banana to the monkey.

4 They further report that significantly more looks targetted what they labelled ‘contrast’ entities, namely ‘the man’s coffee (as well as on the set of contrasting elements such as the teapot taken as a whole)’ (Gennari et al., 2005, p. 256), in Marked Stress than in Neutral Stress. Unfortunately, it is unclear whether the combined looks to ‘contrast entities’ includes looks to ‘the man’s milk’, which is a crucial object for determining the truth value of the sentence *The mother only gave some milk to the* boy. Whether or not the ‘man’s milk’ is included in what Gennari et al. (2005) called ‘contrast entities’, it is difficult to interpret the increased looks to these entities in the Marked Stress condition. It is unexpected in the light of the semantics outlined above (see examples 5–7), since neither the man’s coffee nor the woman’s teapot plays any role in either the non-focal or the focal meaning component in the Marked Stress condition. For establishing the truth value of the sentence *The mother only gave some MILK to the boy*, only the boy’s possessions are relevant. At the same time, it is also hard to interpret this increase as manifestation of the pragmatic effect of contrastivity demonstrated by Ito and Speer (2008, 2011). Ito and Speer (2008, 2011) found that looks increase to the target entities when contrastive accent is used appropriately, not to the entities contrasting with the target entity.

5 We remain agnostic as to whether participants would actually verify the truth of the non-focal meaning component. Kim (2008) found that presuppositional meaning is sometimes verified, and sometimes not depending on the task and the saliency of the entities and the grammatical encoding of the elements. Note that if the focal meaning *is* verified, the verification can only take place after the indirect object has been heard.

6 Note that in Experiment 2, the continuation of the sentence with *brandweerman* ‘fireman’ matches the picture in both the ES and LS condition, so our hypothesis predicts the same anticipatory looks targeting the ‘fireman’ in both conditions, with no significant difference between conditions. As can be seen in **Figure 4**, this expectation was met.
