# Supplementary material for: Early Association of Prosodic Focus with alleen ‘only’: Evidence from Eye Movements in the Visual-World Paradigm
Source: Front Psychol. 2016 Mar 11;7:150. doi: 10.3389/fpsyg.2016.00150 (PMC4786575; doi:10.3389/fpsyg.2016.00150)
Supplement: Supplementary file 1 [file Data_Sheet_1.PDF]

frontiers

Supplementary Material

Early association of prosodic focus with *alleen* 'only': evidence from eye movements in the visual-world paradigm

Iris Mulders [1]*, Kriszta Szendrői [2]*

[1] Utrecht Institute of Linguistics OTS, Utrecht University, Utrecht, The Netherlands
[2] UCL Linguistics, Psychology and Language Sciences, University College London, London, United Kingdom

*** Correspondence:** Dr Kriszta Szendrői, University College London, Linguistics, Psychology and Language Sciences, Chandler House, 2 Wakefield Street, London, WC1N 1PF, United Kingdom.
k.szendroi@ucl.ac.uk

Appendix 1
Stimulus properties

Table S1
Relevant phonetic details of example item *Ik heb alleen* SELDERIJ*/selderij aan de brandweerman/* BRANDWEERMAN *gegeven.* 'I only gave CELERY/ celery to the fireman/ FIREMAN'

	Early stress		Late Stress	
	direct object	indirect object	direct object	indirect object
mean pitch (Hz)	220.94	205.02	212.25	223.26
min F0 (Hz)	177.2	157.45	196.38	153.8
max F0 (Hz)	271.7	384.7	231.01	402.8
pitch range (Hz)	94.5	227.25	34.63	249
duration (ms)	615	573	555	640

Table S2
Test conditions, fillers and expected answers

Conditions	Example	Number of items and expected response
Early Stress	Ik heb alleen WORTELTJES aan de Eskimo gegeven.<br>I have only carrots to the Eskimo given<br>'I only gave CARROTS to the Eskimo.'	16 items<br>Experiment 1: YES<br>Experiment 2: YES<br>Experiment 3: NO
Late Stress	Ik heb alleen worteltjes aan de ESKIMO gegeven.<br>I have only carrots to the Eskimo given<br>'I only gave carrots to the ESKIMO.'	16 items<br>Experiment 1: NO<br>Experiment 2: YES<br>Experiment 3: NO
Control with early stress 1	Ik heb alleen KOMKOMMER aan de dokter gegeven.<br>I have only cucumber to the doctor given<br>'I only gave CUCUMBER to the doctor.'	8 items<br>Experiment 1: NO<br>Experiment 2: NO<br>Experiment 3: YES
Control with early stress 2	Ik heb alleen AARDBEIEN aan de politieman gegeven.<br>I have only strawberries to the police officer given<br>'I only gave STRAWBERRIES to the police officer.'	8 items<br>Experiment 1: NO<br>Experiment 2: NO<br>Experiment 3: YES
Control with late stress 1	Ik heb alleen sla aan de INDIAAN gegeven.<br>I have only lettuce to the Indian given<br>'I only gave lettuce to the INDIAN.'	8 items<br>Experiment 1: YES<br>Experiment 2: NO<br>Experiment 3: YES
Control with late stress 2	Ik heb alleen champagne aan de KONINGIN gegeven.<br>I have only champagne to the queen given<br>'I only gave champagne to the QUEEN.'	8 items<br>Experiment 1: YES<br>Experiment 2: NO<br>Experiment 3: YES
ENDE_filler	De matroos en de astronaut hebben AARDAPPELS.<br>'The sailor and the astronaut have potatoes.'	16 items;<br>8 'YES', 8 'NO'
GEEN_filler	Ik heb GEEN sla aan de CLOWN gegeven.<br>I have no lettuce to the clown given<br>'I didn't give any lettuce to the CLOWN.'	16 items;<br>8 'YES', 8 'NO'
NIED_filler	Ik heb niet aan IEDEREEN broccoli gegeven.<br>I have not to everyone broccoli given<br>'I didn't give broccoli to EVERYONE.'	16 items;<br>8 'YES', 8 'NO'
NIEM_filler	Niemand heeft GROENE KOOL gekregen.<br>Noone has green cabbage got<br>'Noone got GREEN CABBAGE.'	16 items;<br>8 'YES', 8 'NO'

Table S3
Durations of auditory segments in milliseconds

**Segment**	***Average Duration***	**Longest Duration**	**Shortest Duration**
Ik heb<br>*I have*	268	307	168
alleen<br>*only*	279	318	226
Direct Object (DO)	600	765	400
aan de<br>*to the*	257	319	173
Indirect Object (IO)	548	677	354
gegeven<br>*given*	608	693	544

Appendix 2
Instruction sheet for participants. English translation.

Thank you for participating in our experiment!

In this experiment you will be shown a number of pictures. In each picture you will see three persons, who each have two plates (one on each side). On some plates you will see food or drinks. You will then hear a recording that describes the people and their foods or drinks.

If you think that the sound recording and the picture match, press the orange button (using your right thumb if you're right handed, left thumb if you're left handed). If you find that the sound recording does not give an accurate description of what you see in the picture, press the purple button (using your other thumb).

During the experiment your eye movements will be recorded. A sticker will be placed on your forehead, the camera will be adjusted, and then we will start to calibrate. During the calibration a red ball with a black dot in it will appear on all the corners of the screen. Look at the dot **until the ball has disappeared**. Do **not** try to anticipate where the ball will appear next. Keep your head as still as possible.

Once the calibration is succesful, you will first see 12 pictures to practise. Next is another calibration, and then the experiment will really start. Each picture is preceded by a miniature calibration; here, too, keep looking at the dot until it has disappeared. Halfway through the experiment there will be at least one other full calibration.

The experiment itself will take about ten minutes. If you want to take a break during the experiment, you can make contact with the experiment leader(s) by pressing the white button on the intercom to your left (on the wall).

If everything is clear, we can start. Good luck!

Appendix 3
Test item list for all experiments

Early stress condition:
1 Ik heb alleen WORTELTJES aan de eskimo gegeven.
I only gave carrots to the Eskimo.
2 Ik heb alleen GROENE KOOL aan de eskimo gegeven.
I only gave green cabbage to the Eskimo.
3 Ik heb alleen BROCCOLI aan de afrikaan gegeven.
I only gave broccoli to the African guy.
4 Ik heb alleen APPELTAART aan de brandweerman gegeven.
I only gave apple pie to the fireman.
5 Ik heb alleen PAPRIKA aan het jongetje gegeven.
I only gave peppers to the little boy.
6 Ik heb alleen CHAMPIGNONS aan het jongetje gegeven.
I only gave mushrooms to the little boy.
7 Ik heb alleen SELDERIJ aan de brandweerman gegeven.
I only gave celery to the fireman.
8 Ik heb alleen RADIJSJES aan de astronaut gegeven.
I only gave raddishes to the astronaut.
9 Ik heb alleen PERZIKEN aan de koningin gegeven.
I only gave peaches to the queen.
10 Ik heb alleen CHAMPAGNE aan de koningin gegeven.
I only gave champagne to the queen.
11 Ik heb alleen BANANEN aan de astronaut gegeven.
I only gave bananas to the astronaut.
12 Ik heb alleen LIMONADE aan de brandweerman gegeven.
I only gave lemonade to the fireman.
13 Ik heb alleen AARDBEIEN aan de politieman gegeven.
I only gave strawberries to the policeman.
14 Ik heb alleen ANANAS aan de politieman gegeven.
I only gave pineapple to the policeman.
15 Ik heb alleen SINAASAPPEL aan de afrikaan gegeven.
I only gave oranges to the African guy.
16 Ik heb alleen CAPPUCCINO aan de astronaut gegeven.
I only gave a cappuccino to the astronaut.

Late stress condition:
1 Ik heb alleen worteltjes aan de ESKIMO gegeven.
I only gave carrots to the Eskimo.
2 Ik heb alleen groene kool aan de ESKIMO gegeven.
I only gave green cabbage to the Eskimo.
3 Ik heb alleen broccoli aan de AFRIKAAN gegeven.
I only gave broccoli to the African guy.
4 Ik heb alleen appeltaart aan de BRANDWEERMAN gegeven.
I only gave apple pie to the fireman.
5 Ik heb alleen paprika aan het JONGETJE gegeven.
I only gave peppers to the little boy.
6 Ik heb alleen champignons aan het JONGETJE gegeven.

I only gave mushrooms to the little boy.

7 Ik heb alleen selderij aan de BRANDWEERMAN gegeven.
I only gave celery to the fireman.

8 Ik heb alleen radijsjes aan de ASTRONAUT gegeven.
I only gave raddishes to the astronaut.

9 Ik heb alleen perziken aan de KONINGIN gegeven.
I only gave peaches to the queen.

10 Ik heb alleen champagne aan de KONINGIN gegeven.
I only gave champagne to the queen.

11 Ik heb alleen bananen aan de ASTRONAUT gegeven.
I only gave bananas to the astronaut.

12 Ik heb alleen limonade aan de BRANDWEERMAN gegeven.
I only gave lemonade to the fireman.

13 Ik heb alleen aardbeien aan de POLITIEMAN gegeven.
I only gave strawberries to the policeman.

14 Ik heb alleen ananas aan de POLITIEMAN gegeven.
I only gave pineapple to the policeman.

15 Ik heb alleen sinaasappel aan de AFRIKAAN gegeven.
I only gave oranges to the African guy.

16 Ik heb alleen cappuccino aan de ASTRONAUT gegeven.
I only gave a cappuccino to the astronaut.
